# Parkinson's Disease Prevalence and Proximity to Agricultural Cultivated Fields

**DOI:** 10.1155/2015/576564

**Published:** 2015-08-18

**Authors:** Maayan Yitshak Sade, Yair Zlotnik, Itai Kloog, Victor Novack, Chava Peretz, Gal Ifergane

**Affiliations:** ^1^Clinical Research Center, Soroka University Medical Center, 84101 Beer Sheva, Israel; ^2^Faculty of Health Sciences, Ben Gurion University, 84101 Beer Sheva, Israel; ^3^Department of Neurology, Soroka University Medical Center, 84101 Beer Sheva, Israel; ^4^Department of Geography and Environmental Development, Faculty of Humanities and Social Sciences, Ben Gurion University, 84101 Beer Sheva, Israel; ^5^Sackler Faculty of Medicine, School of Public Health, Department of Epidemiology, Tel Aviv University, 6997801 Tel Aviv, Israel

## Abstract

The risk for developing Parkinson's disease (PD) is a combination of multiple environmental and genetic factors. The Negev (Southern Israel) contains approximately 252.5 km^2^ of agricultural cultivated fields (ACF). We aimed to estimate the prevalence and incidence of PD and to examine possible geographical clustering and associations with agricultural exposures. We screened all “Clalit” Health Services members in the Negev (70% of the population) between the years 2000 and 2012. Individual demographic, clinical, and medication prescription data were available. We used a refined medication tracer algorithm to identify PD patients. We used mixed Poisson models to calculate the smoothed standardized incidence rates (SIRs) for each locality. We identified ACF and calculate the size and distance of the fields from each locality. We identified 3,792 cases of PD. SIRs were higher than expected in Jewish rural localities (median SIR [95% CI]: 1.41 [1.28; 1.53] in 2001–2004, 1.62 [1.48; 1.76] in 2005–2008, and 1.57 [1.44; 1.80] in 2009–2012). Highest SIR was observed in localities located in proximity to large ACF (SIR 1.54, 95% CI 1.32; 1.79). In conclusion, in this population based study we found that PD SIRs were higher than expected in rural localities. Furthermore, it appears that proximity to ACF and the field size contribute to PD risk.

## 1. Introduction

Parkinson's disease (PD) is a common neurodegenerative disease characterized by dopaminergic neuron loss. The risk for developing PD is a combination of multiple environmental and genetic factors, and the exact etiology is still unknown [1]. An estimated prevalence and incidence of PD in Israel have been recently assessed using an algorithm based on computerized drug purchase data [2].

A number of studies found PD geographic clustering suggesting a common environmental factor [3, 4]. Exposure to environmental factors such as living in a rural area, well water use, exposure to pesticides, and heavy metals may serve as a risk factor for developing PD [5]. In Israel, clustering of PD patients in agricultural settlements was reported two decades ago [6].

Evidence on the association of pesticides exposure and PD development is controversial [7–11]. In a series of studies, a group of researchers from California found positive associations of pesticides exposure and PD [12–15]; Wang and colleagues, for instance, found higher risk for PD (OR 1.86, 95% CI 1.09; 3.18), associated with combined exposure to ziram, maneb, and paraquat [15]. Other studies found no evidence for pesticides as a risk factor of PD [8, 11].

Consequently, Li et al., in their review, concluded that the current epidemiologic data cannot provide evidence that supports a causal association between pesticide exposure and PD [16]. Furthermore, since PD develops after years of exposures to variety of chemicals, the identification of a specific neurotoxic chemical is extremely difficult [17].

As of 2014, there is no PD registry for the Negev. The present study is based on the prescription database of the largest Health Maintenance Organization (HMO) in Israel—Clalit Healthcare Services. Our aims were to estimate the prevalence and incidence of PD in the Negev population over the past decade and to examine possible geographical clustering of PD patients in the Negev, in conjunction with agricultural exposures.

## 2. Methods

### 2.1. Source Population

We screened all Clalit Health Services (CHS) Health Maintenance Organization (HMO) members in the Negev between the years 2000 and 2012. Approximately 70% of the residents in the Negev are insured by CHS, which owns the only hospital providing neurological care in the region. The remaining 30% of the residents are insured by one of the other three HMOs. Except for higher mean age of the CHS members, the characteristics of all HMO members are similar [18]. This unique setup of the medical system allows us to reduce the potential bias in outcome definition and to include a wide range of the population.

### 2.2. CHS Database

Individual demographic, clinical, laboratory, and medication prescription data of CHS members are fully computerized and were available for our study period. The medication database contains the purchase date, the brand and generic name of the purchased medication, the dose schedule, and the dose dispensed.

### 2.3. PD Assessment

To identify PD patients, we used a refined medication tracer algorithm previously developed and validated by Chillag-Talmor et al. [2]. In brief, we identified all CHS members who purchased at least one of the following anti-Parkinsonian drugs (APD) in the study period: levodopa and levodopa derivatives, amantadine derivatives, dopamine agonists, monoamine oxidase (MAO) B inhibitors, or other dopaminergic agents. Subjects younger than 20 years or older than 84 years of age at first recorded purchase were excluded. We defined PD at three levels of accuracy: definite, probable, and possible based on the follow-up time, the purchase intensity, the combination of drugs purchased, and the age at first recorded purchase [2].

### 2.4. Study Population

The Negev comprises over 55% of the country of Israel's landmass. Approximately 252.5 km^2^ of the land in the Negev is agricultural crop areas, containing mostly field crops [19]. As of 2011, it is home to some 1,121,600 people (or 8.2% of Israel's population), of whom 20% are Bedouin Arabs. The Jewish population in the Negev is older, with a median age of 31 years compared to median age of 15 years among the Bedouin population [20].

Localities were identified as Jewish or Bedouin and as rural or urban based on the definitions of the Central Bureau of Statistics [21]. The majority of the Jewish population resides in cities and about 10% resides in rural localities [20]. Bedouins are in transition period from a seminomad to a stationary life style with approximately 40% living in unrecognized temporary localities [22]. They live in shacks or tents without access to municipal infrastructure such as running water, sewage treatment, or garbage disposal. These localities are not formally registered in the Central Bauru of Statistics databases and therefore are not represented in the map.

### 2.5. Land Use Data

The spatial resolution used in the study corresponds to the center of each locality in Southern Israel and was linked to each subject place of residence. We used the Central Bureau of Statistics database of land use in Israel to identify ACF. Data was gathered from several government offices and private sectors to define types of land use in Israel (e.g., cultivated fields, open space, residence, and industry) [23, 24].

### 2.6. Statistical Analysis

Results are presented in summary tables where normally distributed continuous variables are presented as mean ± SD (standard deviation); not normally distributed continuous variables are presented as medians with minimal and maximal values; and categorical variables are presented as proportions of nonmissing values.

### 2.7. PD Morbidity Rates

#### 2.7.1. Incidence and Prevalence

For the calculation of all the PD rates, PD cases included definite, probable, and possible cases. Incidence cases were defined based on first purchase of APD. Prevalence cases were subjects that purchased APD during the year. Incidence and prevalence rates were calculated from the year 2001 to exclude those who may have been treated prior to study initiation. For the calculation of these rates, we used the total number of CHS members in the appropriate age and gender category (>20 years of age).

#### 2.7.2. Standardized Incidence Rate (SIR)

To address the large random component that may affect disease rates across small areas [25], we used a mixed Poisson model to calculate the smoothed SIR for each locality [26]. Observed cases were incidence cases in each locality. Due to low incidence rates in the rural localities, annual cases were aggregated into three segments of four to five years for the SIR calculations. Expected cases were the number of patients expected in each locality given the total incidence rate in the Negev and the total adult (>20 years of age) population in each locality. Models were adjusted for the percent of elderly (>65 years) and the percent of males in each locality [27].

### 2.8. SIR and ACF Characteristics

To assess the variation in incidence rates with respect to the ACF characteristics located in proximity to each locality, we choose two methods of analysis: (1) we assessed the correlation between the distance from the ACF and the SIR using Spearman test; (2) we assessed the ACF area in the locality surroundings. We used ArcInfo 10.2 to calculate the size of each field and the distance of the fields from each locality. ACF area was calculated in squared kilometers. To define proximity, we set a threshold of 100 meters to allow the inclusion of sufficient area of ACF on one hand and to limit the distance from the locality and therefore reduce the exposure misclassification error as much as possible on the other hand. Localities with no ACF within 100 meters were considered as a reference group, and all other localities were divided into four groups by quartiles.

We used Mann-Whitney and Kruskal-Wallis tests for the comparison of PD morbidity rates by gender, by the type of locality, and by the amount of ACF area located in proximity to the locality.

The study was approved by the SUMC IRB committee. Analyses were performed in SAS 9.4 (SAS Institute Inc., Cary, NC, USA).

## 3. Results

We identified 3,792 cases of PD, of whom 2,231(58.83%) were definite, 647 (17.06%) were probable, and 914 (24.10%) were possible cases. The median age at diagnosis was 73.6 years and 51.7% of the subjects were males (Table 1). The patients reside in 139 localities in the Negev area: 14 Jewish cities (84.7% of the patients), 114 rural Jewish localities (12.5% of the patients), 7 Bedouin permanent localities (2.45% of the patients), and 4 Bedouin temporary settlements (0.35% of the patients) (Table 1).

**Figure 1:**
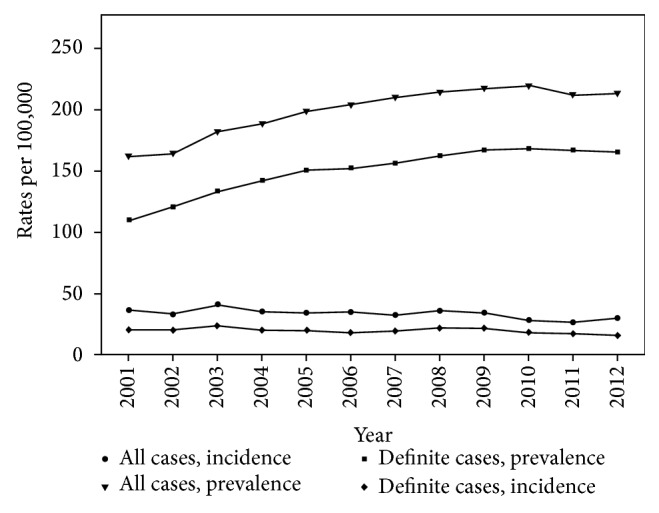
The figure shows the prevalence and incidence rates per 100,000 by year, between the years 2001 and 2012. Prevalence and incidence rates are presented for all cases and definite cases separately.

PD incidence rate ranged between 7.8 and 13.8 cases per 100,000 adults younger than 65 years and between 132.5 and 197.9 cases per 100,000 adults aged 65 years or older, in the years 2001–2012. Annual incidence and prevalence rates were higher among men, mostly in older patients (Table 2). Prevalence rate has increased over the years, while incidence rate remained constant (Figure 1).

We compared patients' characteristics to identify potential confounders that differ significantly by the type of locality. We found that PD residents in Bedouin permanent localities were significantly younger and that the percent of males in these localities was lower (Table 3).

**Figure 2:**
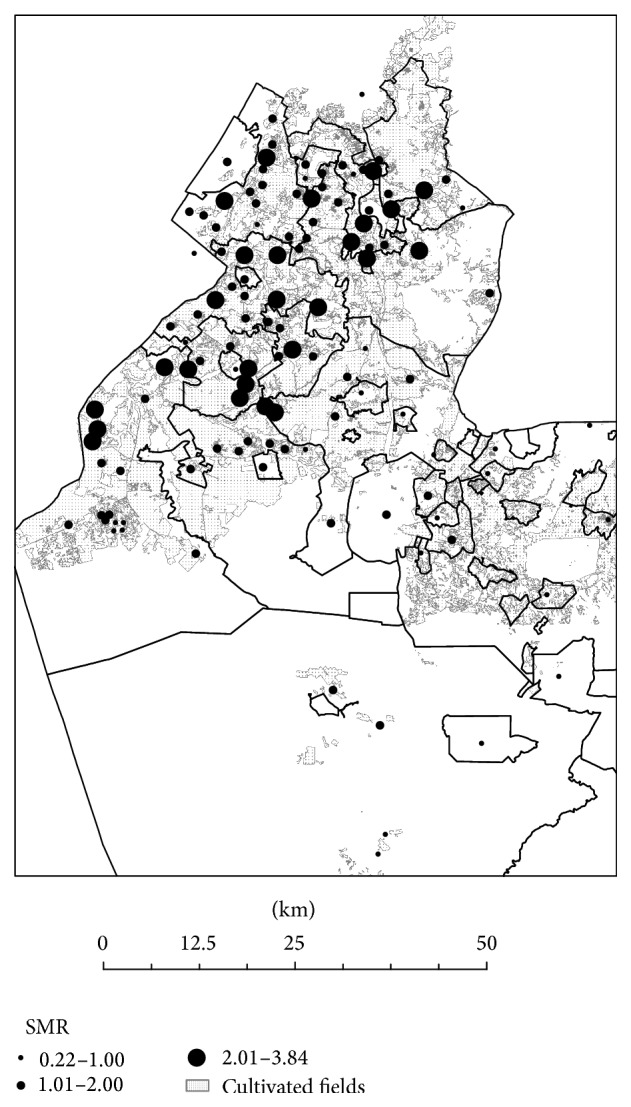
The figure shows the age and gender adjusted standardized incidence rates (SIRs) of Parkinson's disease in localities in Southern Israel. The full lines represent the borders of the southern district in Israel. The graduated symbols represent the SIR in each locality. Agricultural cultivated fields are highlighted in the map.

Figure 2 shows a significant variation in SIR between different localities in the Negev. Median rates were higher than expected in Jewish rural localities, where the median values (and 95% CI) ranged between 1.41 (1.28; 1.53) and 1.62 (1.48–1.76). Among Bedouin localities, both temporary and permanent, SIR was lower than expected (Table 3).

Given the higher SIR observed among rural Jewish localities, we examined the association between the SIR value and the characteristics of the ACF located in proximity to these localities. We found a significant negative correlation between the SIR value and the distance from the nearest ACF (*r* = −0.35, *P* < 0.001).

The quartiles of the ACF area within 100 meters from the localities were as follows: *Q*_1_: 130 km^2^, *Q*_2_: 636 km^2^; and *Q*_3_: 1,612 km^2^. When stratifying Jewish rural localities by the quartiles of ACF area in the locality surroundings, we observed higher SIR values (*P* < 0.01) in localities located in proximity to large ACF (median SIR [95% CI]): 1.37 [1.16; 1.62] in localities with no ACF within 100 meters; 1.41 [1.10; 2.20] in localities with less than 130 km^2^ within 100 meters; and 1.54 [1.32; 1.79] in localities with more than 130 km^2^ of ACF within 100 meters. Estimates of the localities with more than 130 km^2^ (the 1st quartile) were similar and were therefore combined into one category.

## 4. Discussion

In this population based study we found that PD morbidity rates were higher than expected in Jewish rural localities compared to Bedouin localities and Jewish cities. Furthermore, we observed higher SIR values in Jewish localities located in proximity to large agricultural cultivated fields. It appears that proximity to agricultural cultivated fields and the field size contribute to PD risk.

Numerous studies have tried to address the contribution of possible risk factors such as farming, pesticides exposure, living in rural areas, and well water drinking, to PD epidemiology. Similar to our study, the absence of validated exposure assessment is a potential source of bias in most studies [1]. In our study, however, we were able to map the CHS insured PD patients in the entire Negev area and to estimate PD morbidity rates with respect to size of the near ACF, providing another angle of evidence for environmental risk factors for developing PD.

A major strength of this study is the completeness and accuracy of medical and pharmacy data allowed us to perform a large scale epidemiological study. Pharmacy purchase databases are a highly valid source of drug utilization in populations with a universal drug benefit. They are very accurate and closely monitored, since they are maintained for administrative purposes. Thus, pharmacy databases enable observational studies of large populations with long follow-up, reduced selection bias, and increased generalizability [28, 29]. In this study, we used a unique drug tracer algorithm for PD assessment, recently published by Chillag-Talmor et al., which demonstrated a very high sensitivity (96%) and a reasonable rate of false identification of other movement disorders as PD [2]. We chose to base our data on the prescription database of CHS, as it is the largest HMO in Israel, which insures approximately 70% of the population of 730,000 residents in the Negev.

Similar to a former study conducted in Israel, incidence rates remained stable across the study period and prevalence rates had increased over time [2]. The increase in prevalence rate can be explained by a combination of factors: the long duration of PD; general increase in longevity; and possible improvement in medical care expressed in actual increase in disease duration. Our study strengthens the evidence for an increment of PD prevalence, and this finding may have a major impact on medical policy.

Incidence and prevalence rates observed in our study are similar to a previous study conducted in Israel [2], but rather high compared to other countries. In previous reports in other developed countries, rate ranges were 60–350/100,000 for prevalence and 5–26/100,000 for incidence [30–34].

Our SIRs for different localities offer a demonstrative example of the interplay between genetics/ethnicity and environment in PD epidemiology: overall, the median rates were higher in Jewish localities compared to Bedouin. PD prevalence rates among Arabs (~10% of Israel's population > 50 years) were also suggested to be lower compared to Jews in former studies [35], while on the other hand among Ashkenazi Jews rates may be higher, due to the high frequency of PD-associated mutations in LRRK2 (G2019S) and GBA genes among this population in Israel [36, 37]. However, SIRs were higher in both Jews and Bedouins living in rural localities compared to urban people of the same ethnicity, providing further evidence for environmental risk factors for developing PD.

Our study had a few limitations. First, we did not have data regarding the residential history of the patients or the characteristics of the ACS located in proximity to the localities (i.e., pesticides use, type of crops), which may have resulted in exposure misclassification. However, since the study population comprises mostly elderly people, we believe it is safe to assume that most patients did not change their place of residence in the recent decades. Second, we did not have data on the patients' origin and therefore we were not able to properly identify Ashkenazi Jewish origin, a known genetic risk factor for PD. Third, patients who were not insured by Clalit HMO were not included in our analysis. Despite that, because Clalit insures the majority of the Negev population, we believe that our study population is representative of the total population in the Negev. In addition, Bedouin or Jewish origin was assigned based on the place of residence. Lastly, since we identified PD patients based on medications purchase, our definition may be affected by population behavior and compliance to treatment. Given that limitation, we address the morbidity rates found in the Bedouin population (characterized with low SES and low compliance for treatment) with caution.

In conclusion, our data serves as a demonstrative example and provides further evidence for the interplay between ethnicity and environmental factors contributing to PD epidemiology. Rural areas and proximity to large ACF seem to play a role in PD according to our approach of stratification, and further studies are warranted in order to further elucidate this issue.

## Figures and Tables

**Table 1:** Population characteristics.

Population characteristics	*N* = 3792

Parkinson's disease, % (*n*)	
Definite	58.83 (2,231)
Probable	17.06 (647)
Possible	24.10 (914)

Male gender, % (*n*)	51.71 (1,961)

Family status, % (*n*)	
Single	6.15 (198)
Married	79.21 (2,552)
Divorced	4.81 (155)
Widow	9.84 (317)

Age at diagnosis	
Median (min; max)	73.6 (20.11; 84.48)

Type of locality, % (*n*)	
Jewish city (14 localities)	84.70 (3,212)
Jewish rural agricultural locality (114 localities)	12.53 (475)
Bedouin Arab permanent locality (7 localities)	2.45 (93)
Bedouin Arab temporary settlement (4 localities)	0.32 (12)

**Table 2:** Annual prevalent and incident PD cases by gender, 2001–2012.

Year	Incidence rate per 100,000 adults			Prevalence rate per 100,000 adults		
	Men	Women	Total	Men	Women	Total
Age < 65						
2001	12.3	7.9	10.0	47.1	28.4	37.4
2002	7.2	8.9	8.0	46.9	33.0	39.7
2003	13.2	14.2	13.8	57.4	41.7	49.3
2004	6.2	11.6	9.0	58.4	48.0	53.0
2005	11.2	13.3	12.3	60.6	55.6	58.0
2006	9.9	13.2	11.6	63.8	59.4	61.6
2007	9.7	8.5	9.1	67.0	61.6	64.2
2008	10.3	10.8	10.6	70.7	63.0	66.8
2009	13.0	12.3	12.7	77.7	66.3	71.8
2010	10.69	8.3	9.5	79.1	66.8	72.8
2011	9.78	6.3	7.9	81.7	64.7	73.0
2012	10.3	6.5	7.8	85.3	62.0	73.4

Age ≥ 65						
2001	209.9	167.7	185.5	1054.8	740.9	873.8
2002	190.7	166.5	176.8	1002.7	774.0	870.8
2003	261.7	151.0	197.9	1115.5	807.6	937.9
2004	229.3	150.3	183.6	1136.5	826.8	957.2
2005	184.4	141.5	159.6	1162.9	865.4	990.7
2006	212.3	131.2	165.5	1186.8	878.7	1008.4
2007	231.3	122.2	168.2	1257.5	880.4	1039.6
2008	213.0	161.5	183.3	1300.1	897.2	1067.6
2009	207.1	126.2	160.6	1271.5	910.2	1063.6
2010	172.3	117.5	140.8	1279.2	926.1	1076.7
2011	162.8	109.9	132.5	1231.6	860.3	1019.3
2012	193.5	131.7	158.2	1232.3	846.8	1012.0

This table shows the annual incidence and prevalence rates. Total rates and rates stratified by age and gender are presented per 100,000 people between the ages 25 and 85 in Southern Israel. Prevalence cases were subjects that purchased APD during the year. Incidence cases for each year were assigned based on first purchase.

**Table 3:** Age and gender adjusted standardized incidence rates (SIRs) of Parkinson's disease and population characteristics, by the type of locality.

	Type of locality				*P* value
	Bedouin, temporary (*n* = 4)	Bedouin, permanent (*n* = 7)	Jewish, rural (*n* = 114)	Jewish, city (*n* = 14)	
PD accuracy, % (*n*)					
Definite	50 (6)	33.33 (31)	62.74 (298)	59 (1,896)	
Probable	8.33 (1)	9.68 (9)	15.16 (72)	17.6 (565)	
Possible	41.66 (5)	56.99 (53)	22.11 (105)	23.4 (751)	
Total	100 (12)	100 (93)	100 (475)	100 (3,212)	

^*∗*^SIR					
Median (95% CI^*∗∗∗*^)					
2001–2004	—	0.28 (0.21; 0.30)	1.41 (1.28; 1.53)	1.02 (0.87; 1.16)	<0.001
2005–2008	0.46 (0.36; 0.55)	0.27 (0.22; 0.31)	1.62 (1.48; 1.76)	1.01 (0.85; 1.17)	<0.001
2009–2012	0.47 (0.38; 0.62)	0.29 (0.23; 0.33)	1.57 (1.44; 1.80)	1.04 (0.89; 1.20)	<0.001

^*∗∗*^Male gender, % (*n*)	50 (6)	42 (39)	48.42 (230)	52.5 (1,686)	0.093

^*∗*^Age at diagnosis					
Median (min; max)	70.8 (55.6; 76.8)	58.5 (20.9; 84.1)	71.7 (21.3; 84.5)	74 (20.11; 84.48)	<0.001

^*∗*^ *P* values were obtained from Kruskal-Wallis test.

^*∗∗*^ *P* values were obtained from chi-square test.

^*∗∗∗*^95% confidence intervals calculated as distribution-free confidence limits.
